# Opposing Responses of Bird Functional Diversity to Vegetation Structural Diversity in Wet and Dry Forest

**DOI:** 10.1371/journal.pone.0164917

**Published:** 2016-10-14

**Authors:** Holly Sitters, Alan York, Matthew Swan, Fiona Christie, Julian Di Stefano

**Affiliations:** School of Ecosystem and Forest Sciences, University of Melbourne, Creswick, Victoria, Australia; University of Sydney, AUSTRALIA

## Abstract

Disturbance regimes are changing worldwide, and the consequences for ecosystem function and resilience are largely unknown. Functional diversity (FD) provides a surrogate measure of ecosystem function by capturing the range, abundance and distribution of trait values in a community. Enhanced understanding of the responses of FD to measures of vegetation structure at landscape scales is needed to guide conservation management. To address this knowledge gap, we used a whole-of-landscape sampling approach to examine relationships between bird FD, vegetation diversity and time since fire. We surveyed birds and measured vegetation at 36 landscape sampling units in dry and wet forest in southeast Australia during 2010 and 2011. Four uncorrelated indices of bird FD (richness, evenness, divergence and dispersion) were derived from six bird traits, and we investigated responses of these indices and species richness to both vertical and horizontal vegetation diversity using linear mixed models. We also considered the extent to which the mean and diversity of time since fire were related to vegetation diversity. Results showed opposing responses of FD to vegetation diversity in dry and wet forest. In dry forest, where fire is frequent, species richness and two FD indices (richness and dispersion) were positively related to vertical vegetation diversity, consistent with theory relating to environmental variation and coexistence. However, in wet forest subject to infrequent fire, the same three response variables were negatively associated with vertical diversity. We suggest that competitive dominance by species results in lower FD as vegetation diversity increases in wet forest. The responses of functional evenness were opposite to those of species richness, functional richness and dispersion in both forest types, highlighting the value of examining multiple FD metrics at management-relevant scales. The mean and diversity of time since fire were uncorrelated with vegetation diversity in wet forest, but positively correlated with vegetation diversity in dry forest. We therefore suggest that protection of older vegetation is important, but controlled application of low-severity fire in dry forest may sustain ecosystem function by enhancing different elements of FD.

## Introduction

Accelerating rates of environmental change present challenges to conservation practitioners worldwide [1,2]. The effectiveness of conservation initiatives is often measured by species diversity metrics, but focus is currently shifting to other aspects of diversity such as functional, genetic and phylogenetic, which have the capacity to reveal processes underlying patterns in community composition [3–5]. Functional diversity (FD) measures the range, abundance and distribution of species’ traits such as body mass, feeding guild and nest type, and links species diversity with ecosystem function [5–7]. FD can be related to ecosystem resilience because systems are more likely to absorb shocks, reorganise and retain their initial structure if they support several species that perform the same function but differ in their responses to disturbance [8,9]. Identification of the key drivers of FD can therefore provide insight into the vulnerability of ecosystems to environmental change [10].

In theory, spatially variable environments offer more opportunities for resource partitioning and are thus expected to support greater FD [11]. To date, most studies of FD-environment relationships have used map-based measures of environmental variation such as land-use type [12,13], landscape context [14,15] and gradients in topography and climate at regional scales [10,16]. Birds, for example, provide a range of ecosystem functions including pollination and pest regulation [17], and bird FD has been positively associated with land-cover-type diversity [18]. While support for theorised positive associations between FD and landscape diversity is emerging from studies that recognise categorical habitat types, there is a lack of information regarding responses of FD to continuous habitat measures [19,20]. Large-scale studies based on categorical habitat types help agencies prioritise conservation efforts at global and national levels [21], but management actions are normally applied at local scales. Identification of FD drivers at local scales is therefore needed to guide conservation management [22], and ultimately foster resilient ecosystems.

Increasingly, planned fire is used as a management tool to reduce the risk of large, intense wildfires and conserve biodiversity [23–25]. Ecological fire management is often guided by an assumption that different species require resources provided by different vegetation age classes (times since fire), and to this end managers seek to maintain a mosaic of younger and older vegetation [26–28]. However, older vegetation is of disproportionate importance to faunal diversity in some regions [29,30], and might thus be expected to support greater FD. Responses of faunal FD to fire are little-studied but two site-based studies indicate that responses of bird FD to vegetation age vary with fire severity [31,32], which describes spatial patterns of vegetation damage [33]. FD of understory birds in Amazonian forest was unrelated to high-severity fire frequency categories [32], whereas negative responses of FD to time since fire were identified in southeast Australia where recent fire was of low severity, suggesting that patchy fire generates resource diversity [31]. These studies identify relationships between FD and fire using data from specific points (sites) in the landscape, but it is rarely feasible to manage fire at this scale. Use of landscape sampling units to examine relationships between FD, vegetation age and resource diversity at management-relevant scales will reveal how planned fire may be used to sustain FD [34].

Fire can directly cause mortality of individuals with particular traits [35] but our focus is on how it affects FD indirectly by altering resource diversity. A key element of resource diversity to which fauna respond is vegetation structural diversity, which describes the vertical and horizontal distribution of vegetation [36]. The extent to which fire influences vegetation diversity is a function of both time since fire and fire severity; high-severity fire simplifies vegetation structure in the short term, while low-severity fire often only removes understory vegetation in tall forest, and can create a patchwork of burnt and unburnt vegetation [23]. In principle, fire influences FD indirectly by altering vegetation structure, and relationships between fire, vegetation diversity and FD are driven by multiple associations between individual traits and individual attributes of vegetation structure.

The direction and strength of relationships between FD and vegetation diversity are expected to vary with climate or productivity; responses of bird species diversity to forest management actions have been shown to differ along productivity gradients because the effects of vegetation structure are a function of available energy [36,37]. In highly productive systems with long periods between disturbances, a few species can become dominant and species diversity decreases as vegetation diversity increases [38]. In contrast, positive relationships between vegetation diversity and bird species diversity are expected in less productive systems characterised by frequent disturbance, which prevents competitive dominance. The influence of productivity on relationships between FD and vegetation diversity is unclear, but it is likely that vegetation diversity and productivity interact to influence FD [10]. Testing for these interactions will provide a basis for effective fire management in areas of differing productivity.

Our primary aim was to investigate relationships between FD and vegetation diversity in dry (low productivity) and wet (high productivity) forest. Our study area spanned a 70-year chronosequence in time since fire in the Otway Ranges, southeast Australia, where we used 36 landscape sampling units to test two hypotheses. First, we predicted a positive association between FD and vegetation diversity in dry forest where fire is frequent. Second, we anticipated a negative relationship between FD and vegetation diversity in wet forest subject to infrequent fire. In addition we examined relationships between vegetation diversity and time since fire to determine whether time since fire is a useful surrogate for vegetation diversity, and consequently FD. Collectively, results will help to elucidate the key environmental drivers of FD in southeast Australian forest systems, and guide ecological fire management in different forest types.

## Methods

### Study area

**Fig 1:**
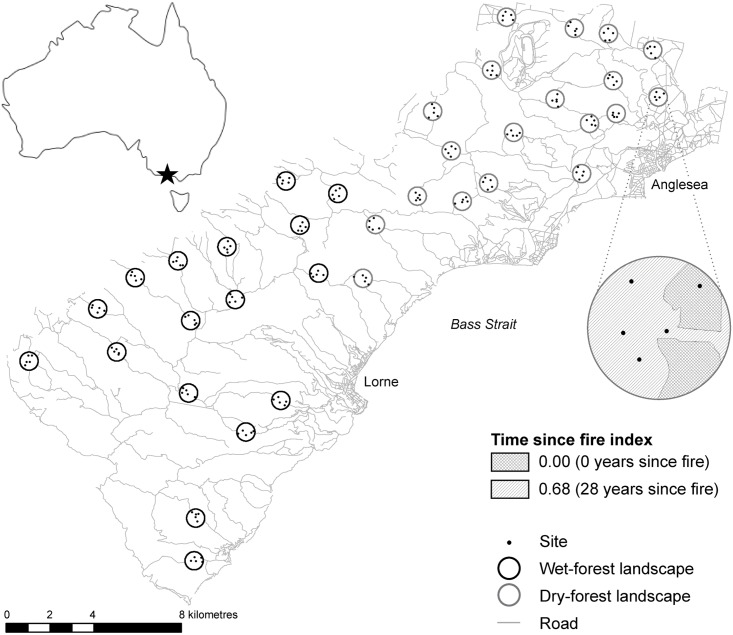
Location of the study area in southeast Australia. The enlarged detail shows a single dry-forest landscape sampling unit of 100 ha (1.13 km diameter). Modified from Sitters et al. [52] under a CC BY license, with permission from Elsevier Ltd., original copyright 2014.

The study area covered 59,000 ha of the Otway Ranges (Great Otway National Park and Forest Park) in southeast Australia (Fig 1), where the climate is mild (mean annual minimum and maximum temperatures are 10.5°C and 18.2°C), and mean annual rainfall ranges from 661 mm in the northeast to 1259 mm in the southwest [39]. Heathlands of low, dense shrubs in the northeast of the study area merge with heathy woodlands at low elevations (30–279 m above sea level (a.s.l)), and at higher elevations further southwest (200–600 m a.s.l), complex topography supports tall-open eucalypt forest. We used ArcMap [40] to classify two broad forest types: wet and dry. Dry forest includes the heathland and tall-mixed woodland ecological vegetation divisions (EVDs) [41], and has a low canopy (<30 m). Wet forest reaches 50–60 m in height and comprises the foothills forest, forby forest and moist forest EVDs. In both forest types, adaptations such as thick bark, epicormic shoots and regeneration from lignotubers enable most trees to survive fire, but severe fire can kill trees in some instances [42].

Time-since-fire maps of the study area were derived from individual fire-history layers corresponding to every year since 1939. Large wildfires affected the study area in 1939 and 1983, and planned fire has been applied increasingly frequently since 2008. Planned burns are normally implemented such that 30–70% of vegetation remains unburnt [43]. Severe wildfire often consumes canopy vegetation, while low-severity planned fire consumes understory and midstory vegetation but rarely reaches the canopy. We converted time-since-fire values to a time-since-fire index (hereafter TSF) scaled from zero (freshly burnt) to one (senescence) [41,44], to account for different rates of post-fire vegetation development in the two forest types. For example, dry forest typically reaches maturity 8 years after fire, whereas wet forest does not reach maturity until 26 years post-fire [41]. The mean and range of TSF index values were similar in both forest types: in dry forest the mean TSF index was 0.56 (range 0.00–0.80), which corresponds to 21 years since fire (range 0–46 years); in wet forest the mean TSF index was 0.58 (range 0.05–0.77), corresponding to 38 years since fire (range 0.5–73 years).

### Landscape sampling design

Our sampling units were circular landscapes of 100-ha (1.13 km diameter; Fig 1), which corresponded to the scale at which fire management is practised in the region. We selected sampling-unit centres from 500 random points, and stratified by (i) forest type, and (ii) areas that were and were not prioritised for planned burning to ensure adequate coverage of younger vegetation. Centre points were positioned >3 km apart, and >3 km from urban areas where planned burning is particularly frequent. In each forest type, we ensured that landscapes captured a gradient in the mean and diversity of TSF, from homogeneous landscapes of uniform TSF, to landscapes containing patches of up to five TSF values. Thirty-two landscapes were established in year one (2010) and four more were established in year two (2011), resulting in a total of 36 sampling units, 19 in dry forest, and 17 in wet forest.

Within each landscape, we positioned five sites using a restricted random protocol, ensuring that at least one site was placed in patches of each TSF represented in the landscape. We established a 100-m transect at each site along a random bearing, ensuring that transects were >200 m apart and did not extend to within 50 m of roads or neighbouring patches.

### Bird surveys

Birds were surveyed by four trained observers using two 10-minute point interval counts at the 20- and 80-m marks of site transects. Birds were recorded as seen, heard or flying over and assigned to a distance category (0–25 m, 25–50 m, 50–100 m or >100 m from the observation point). During each survey year, sites were surveyed twice, once within four hours of sunrise, and once within three hours of sunset. Repeat surveys were undertaken on a different day, and observers were rotated among sites of different forest type, TSF and time of day to reduce the potential influence of observer bias. Surveys were conducted in good weather between September and December, which is the breeding season and is when most summer migrants arrive. Bird surveys were approved by the University of Melbourne School of Land and Environment Ethics Committee (Register Number 1011632.5), and field work was conducted under the National Parks Act (Research Permit Number 10005348) and Forests Act (Scientific Permit Number 10005514).

Landscape-level presence-absence estimates were generated using data from within a 50-m radius of sites. Distance sampling has shown that although the mean probability of detection for species in the study area within 50 m is low (0.49, 95% CI 0.41–0.56), it does not vary significantly among recently-burnt and long-unburnt vegetation [45]. To derive bird response variables at the scale of landscape sampling units, we pooled data from (i) the 20- and 80-m marks of transects, (ii) the five sites in a landscape and (iii) repeat surveys conducted in the same year, to yield two presence-absence estimates per landscape corresponding to the two years’ surveys.

### Landscape functional diversity

Landscape-level presence-absence data were used to derive estimates of landscape species richness and FD. To calculate FD, we focussed on six traits associated with resource acquisition and use, which are expected to influence relationships between species diversity and ecosystem function [46]: body mass, clutch size, food type, foraging habit, foraging location and nest form [31]. We used the six traits to quantify four aspects of FD: richness, evenness, divergence and dispersion [47,48]. Functional richness (FRic) and functional evenness (FEve) are broadly analogous to taxonomic richness and evenness. They are independent of each other and quantify different aspects of the distribution and complementarity of species in a multi-dimensional convex hull volume of functional space occupied by a community [6]. FRic is the volume of functional space occupied by the species in a community, and FEve quantifies the regularity of the species’ distribution in this volume [47]. Functional divergence (FDiv) represents the level of niche differentiation in the assemblage and increases with the number of species that have unique functional trait values; it measures the distribution of species within the convex hull, independent of its volume [47]. We calculated FD using species occupancy data, so FEve and FDiv are interpreted in terms of the relative positions of species in functional space. Functional dispersion (FDis) measures the dispersion of species in functional trait space as the average distance of individual species to the centroid of all species [48], effectively combining FRic and FDiv. We calculated the four indices in the R statistical environment [49] using the package FD [50]. FRic and FDis have no upper limit, and FEve and FDiv range between zero and one. Landscape species richness was moderately correlated with FRic (r = 0.58), but all other variables were uncorrelated (r <0.5).

### Vegetation measurements

We derived indices of vertical and horizontal vegetation structural diversity from measures of four variables [51–53]: lower understory (percent cover 0–0.5 m above ground), upper understory (percent cover 0.5–2.0 m), midstory (percent cover from 2 m up to and including the sub-canopy), and canopy (percent cover of the tallest stratum). Measurements were taken at 3-m intervals along 100-m transects; lower and upper understory were measured as the presence of contacts with a vertical pole, and midstory and canopy were derived from the presence of vegetation on the cross-hairs of a densiometer. We measured structure variables at all sites during year one, and remeasured sites burned <3 years before or after the first field season in year two because young vegetation changes rapidly. The same four vegetation structure variables were used to derive indices of both vertical and horizontal diversity.

### Landscape diversity indices

We combined measurements from the five sites in a landscape to quantify four indices of landscape-scale diversity, two based on vegetation structure, and two based on TSF. Both vertical and horizontal vegetation structural diversity were calculated using Shannon’s diversity index. Vertical diversity measured the extent to which vegetation was concentrated in one layer (low vertical diversity), or evenly distributed among layers (high vertical diversity). It was derived from the landscape means of the four vegetation structure variables, and was analogous to MacArthur and MacArthur’s [53] Foliage Height Diversity. Horizontal diversity measured the extent to which vegetation was clustered on transects (low horizontal diversity) or evenly distributed (high horizontal diversity). It was derived from means of the sub-sampling locations at 3-m intervals. Vertical and horizontal diversity index values were calculated for each landscape in each survey year, and were uncorrelated (r <0.5).

Additionally, we quantified the mean and diversity (Shannon’s diversity index) of TSF per landscape per year. TSF diversity distinguished landscapes that contained sites of five different TSF (low TSF diversity), to homogeneous landscapes of uniform TSF (high TSF diversity. The mean and diversity of TSF were positively correlated (r = 0.7) because homogeneous landscapes tended to be long-unburnt, so we did not use them as predictors in the same statistical models.

### Data analysis

Our analysis involved three stages; first we explored the responses of species richness and FD to vegetation diversity in wet and dry forest. Second, we examined relationships between the occurrence of individual traits and vegetation structure variables, and finally we considered the extent to which the mean and diversity of landscape TSF are related to vegetation diversity.

In stage one, we used linear mixed models (LMM) to investigate responses of species richness and FD to vegetation diversity in wet and dry forest. The mixed-modelling framework accommodated variance associated with the nestedness of the design [54]; landscape was specified as a random effect throughout the analysis to accommodate correlation structure associated with repeat visits to landscapes in the two survey years. We assessed assumptions of normality and homogeneity of variance using graphical methods and modelled all response variables using LMMs with Gaussian errors. Candidate model sets comprised six models: vertical and horizontal diversity alone and in additive and interactive combination with the two-level categorical variable forest type. Models containing additive terms tested for consistent response shapes among forest types, and models containing interactions tested for contrasting responses. Year was specified as an additive fixed effect in all models to ensure estimates associated with other variables were independent of year. We used information-theoretic model selection to rank candidate sets of six models per response variable [55]. Support for models was compared using the small-sample-size adjustment of Akaike’s information criterion, and Akaike weights were calculated to show the relative likelihood that a model was the most parsimonious. We used the R packages lme4 and MuMIn to undertake model selection [56,57]. Models were evaluated using R^2^ as a measure of fit; marginal R^2^ was the variance explained by fixed effects, and conditional R^2^ was the variance explained by both fixed and random effects [58].

Stage two of our analysis involved identifying associations between the occurrence of individual traits and individual vegetation structure variables which are expected to underpin relationships between FD and vegetation diversity. Again, we quantified variables at the landscape scale (bird presence-absence and the mean cover of vegetation structure variables). We used a fourth-corner model that relates species traits to vegetation attributes by fitting a predictive model of species occurrence (L) as a function of matrices of vegetation structure variables (R) and species traits (Q) and their interaction [59,60]. The R-Q interaction shows how the occurrence of traits varies with vegetation structure and generates coefficients that quantify the strength of associations. We used the traitglm function in the R package mvabund [61] to apply multivariate generalised linear models with a binomial distribution, and we used a LASSO approach to simplify variable selection by setting model terms that do not explain any variation to zero [62].

During the final stage of analysis we considered the extent to which the mean and diversity of landscape TSF were related to vegetation diversity. We used the same statistical methods as in stage one; vertical and horizontal diversity were response variables and the mean and diversity of TSF were predictor variables in LMMs. Candidate model sets consisted of each predictor variable alone, and in additive and interactive combination with forest type, for a total of six candidate models. In all models, year was specified as an additive fixed effect, and landscape was specified as a random effect. We compared levels of support for models using information-theoretic model selection, and used marginal and conditional R^2^ to measure fit.

## Results

**Fig 2:**
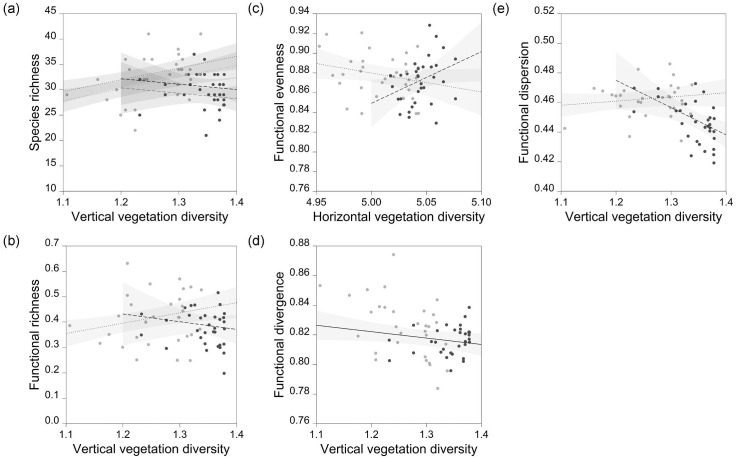
Responses of species richness and functional diversity to vegetation diversity indices. Predictions and 95% confidence intervals are derived from top-ranked linear mixed models. Point colours and line types correspond to different forest types (dry forest = grey points, dotted lines; wet forest = black points, dashed lines). Lines corresponding to year 1 (grey) and year 2 (black) are displayed where year had a significant influence on the response variable.

Responses of species richness and FD to vegetation diversity differed in wet and dry forest, and in general, vertical diversity was a more important predictor than horizontal diversity (Table 1 and Fig 2). Species richness, FRic and FDis were best predicted by an interaction between vertical diversity and forest type; they were positively associated with vertical diversity in dry forest, and negatively related to vertical diversity in wet forest (Fig 2). In contrast, the top-ranked model of FEve contained an interaction between horizontal diversity and forest type; the relationship was negative in dry forest and positive in wet forest. Vertical diversity alone was the best predictor of FDiv, but the negative relationship was weak (Table 1, Fig 2). Interaction terms in other top-ranked models were statistically significant with the exception of the species richness model, which contained a weak negative association in wet forest and a positive response to vertical diversity in dry forest. Species richness was greater in year 2 than year 1, but no FD metrics responded to year.

**Table 1:** Responses of bird species richness and functional diversity to vertical (VD) and horizontal vegetation diversity (HD) in different forest types (FT) and years (Y) derived from linear mixed models.

Response variable	Model		Delta AIC_c_	Akaike weight	Estimate ± 95% CI	*P*	R^2^m	R^2^c
Species richness								
	Y + VD × FT		0.00	0.64			0.29	0.35
		Year 2			1.78 ± 1.65	0.039		
		VD			23.273 ± 12.341	<0.001		
		Wet forest			40.923 ± 48.372	0.100		
		VD × Wet forest			-33.930 ± 36.318	0.072		
	Y + VD + FT		1.22	0.35			0.25	0.34
		Year 2			1.738 ± 1.667			
		VD			19.331 ± 11.917	0.003		
		Wet forest			-4.218 ± 2.347	0.001		
Functional richness								
	Y + VD × FT		0.00	0.41			0.16	0.30
		Year 2			0.023 ± 0.037	0.226		
		VD			0.407 ± 0.298	0.011		
		Wet forest			0.890 ± 1.168	0.138		
		VD × Wet forest			-0.711 ± 0.877	0.116		
	Y + VD + FT		0.33	0.35			0.12	0.29
		Year 2			0.022 ± 0.037	0.247		
		VD			0.324 ± 0.285	0.031		
		Wet forest			-0.057 ± 0.056	0.053		
Functional evenness								
	Y + HD × FT		0.00	0.53			0.12	0.12
		Year 2			0.002 ± 0.011	0.667		
		HD			-0.192 ± 0.234	0.112		
		Wet forest			-3.610 ± 2.821	0.015		
		HD × Wet forest			0.716 ± 0.560	0.016		
	Y + HD		1.95	0.20			0.02	0.07
		Year 2			0.001 ± 0.011	0.848		
		HD			-0.098 ± 0.177	0.281		
Functional divergence								
	Y + VD		0.00	0.29			0.09	0.44
		Year 2			-0.004 ± 0.006	0.173		
		VD			-0.043 ± 0.045	0.067		
	Y + HD		0.82	0.19			0.07	0.44
		Year 2			-0.004 ± 0.006	0.155		
		HD			-0.115 ± 0.138	0.106		
	Y + VD + FT		1.29	0.15			0.11	0.44
		Year 2			-0.004 ± 0.006	0.171		
		VD			-0.027 ± 0.055	0.336		
		Wet forest			-0.006 ± 0.011	0.316		
	Y + HD × FT		1.53	0.14			0.14	0.47
		Year 2			-0.004 ± 0.006	0.241		
		HD			-0.122 ± 0.189	0.206		
		Wet forest			-1.858 ± 2.253	0.109		
		HD × Wet forest			0.368 ± 0.447	0.112		
Functional dispersion								
	Y + VD × FT		0.00	0.85			0.36	0.56
		Year 2			0.004 ± 0.005	0.114		
		VD			0.028 ± 0.046	0.232		
		Wet forest			0.268 ± 0.179	0.005		
		VD × Wet forest			-0.212 ± 0.134	0.004		

Levels of forest type are dry and wet; estimates associated with wet forest represent contrasts with dry, and estimates associated with Year 2 represent contrasts with Year 1. The small-sample-size adjustment of Akaike’s information criterion (AIC_c_) was used to rank models. Models within two units of the top-ranked model are shown with Akaike weights. Parameter estimates with 95% confidence intervals (CI) are displayed with their statistical significance (*P*). Two measures of fit are included: marginal R^2^ (R^2^m) is the variance explained by fixed factors and conditional R^2^ (R^2^c) is the variance explained by both fixed and random factors.

**Fig 3:**
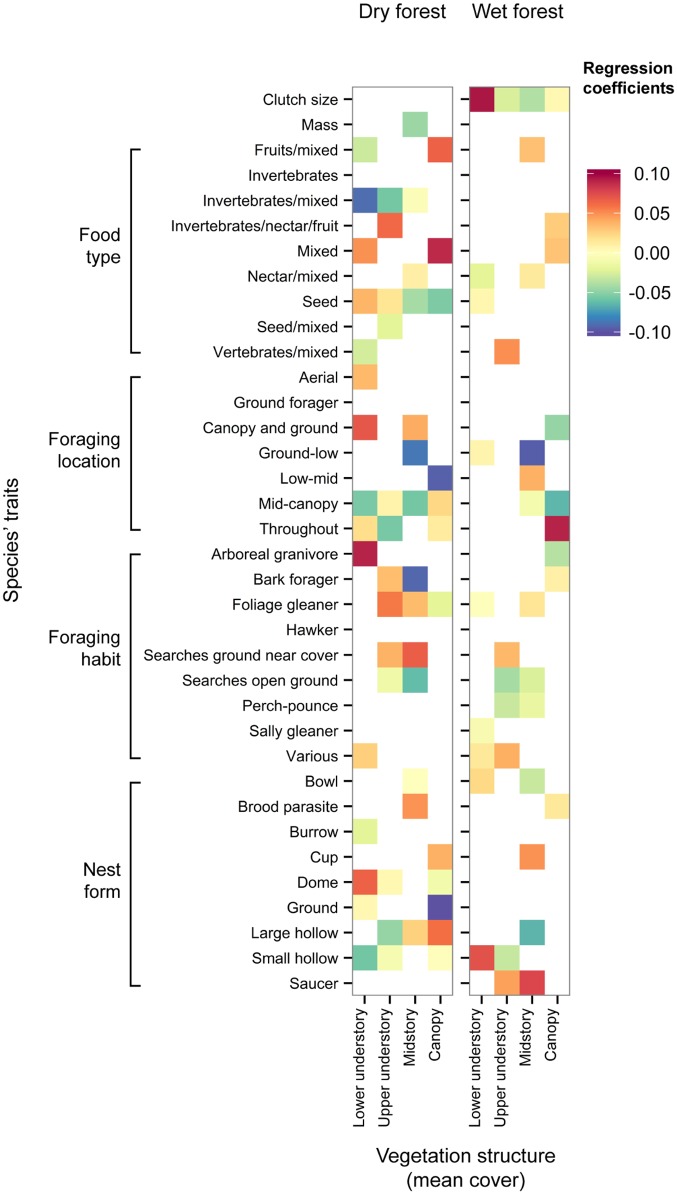
Fourth-corner interaction coefficients for the relationship between bird traits and vegetation structure variables. Statistically significant relationships are indicated in red (positive) and blue (negative); the shade of the colour represents the strength of the association. Clutch size is an ordinal variable, mass is continuous and other trait variables are categorical.

The fourth-corner analysis revealed associations between individual bird traits and vegetation structure variables (Fig 3). Some associations were found in both forest types; for example, we identified a positive relationship between the presence of foliage-gleaning birds and midstory cover, and a negative response of ground-feeding birds to midstory cover. However, most associations between traits and vegetation structure were not common to both forest types, and trait-vegetation associations were generally stronger and more numerous in dry forest.

**Fig 4:**
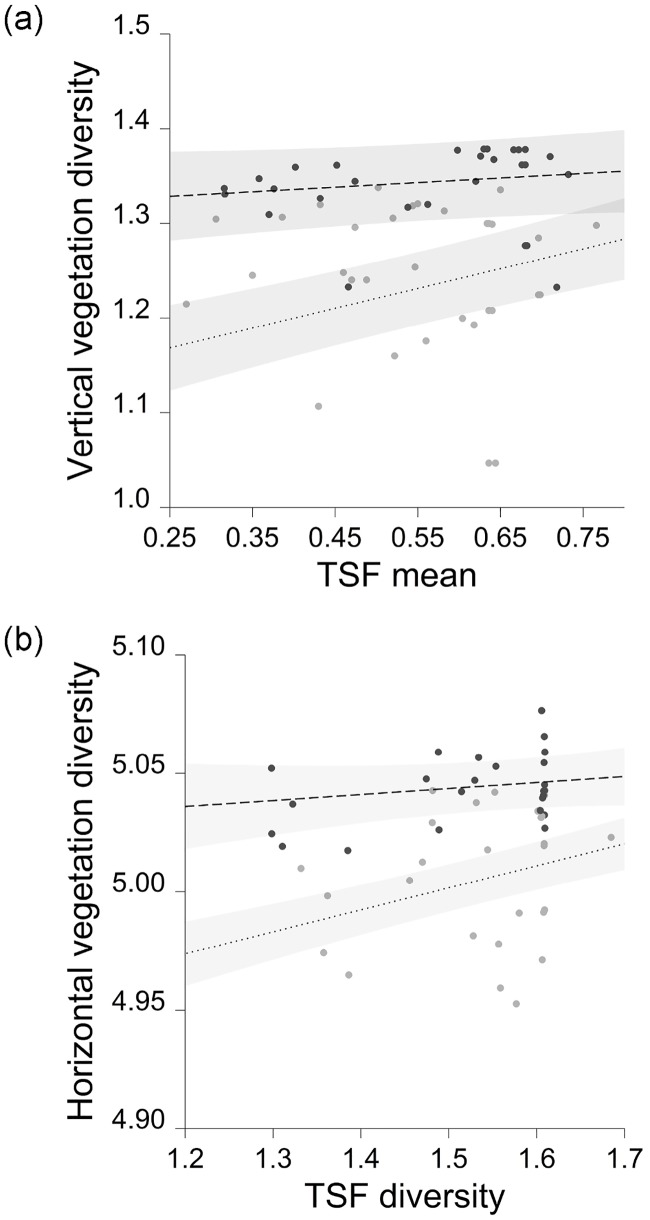
Responses of vertical and horizontal diversity to the mean and diversity of the time since fire index (TSF). Predictions and 95% confidence intervals are derived from top-ranked linear mixed models. Point colours and line types correspond to different vegetation types (dry forest = grey points, dotted lines; wet forest = black points, dashed lines).

Both vertical and horizontal vegetation structural diversity were positively correlated with landscape TSF variables (Fig 4), but relationships were very weak in wet forest. Vertical diversity was best predicted by an interaction between the mean of TSF and forest type, and the top-ranked model of horizontal diversity contained an interaction between the diversity of TSF and forest type (Table 2). Neither of the vegetation diversity variables was related to year.

**Table 2:** Responses of vegetation diversity to the mean (TSF_m_) and diversity (TSF_d_) of the time since fire index in different forest types (FT) and years (Y) derived from linear mixed models.

Model			Delta AIC_c_	Akaike weight	Estimate ± 95% CI	*P*	R^2^m	R^2^c
Vertical diversity								
	Y + TSF_m_ × FT		0.00	0.95			0.35	0.69
		Year 2			-0.001 ± 0.005	0.667		
		TSF_m_			0.207 ± 0.079	<0.001		
		Wet forest			0.201 ± 0.082	<0.001		
		TSF_m_ × Wet forest			-0.162 ± 0.107	0.005		
	Y + TSF_m_ + FT		6.05	0.04			0.34	0.69
		Year 2			-0.001 ± 0.006	0.855		
		TSF_m_			0.120 ± 0.062	<0.001		
		Wet forest			0.110 ± 0.055	<0.001		
	Y + TSF_d_ × FT		15.21	0.00			0.33	0.64
		Year 2			-0.004 ± 0.007	0.201		
		TSF_d_			0.009 ± 0.007	0.021		
		Wet forest			0.112 ± 0.028	<0.001		
		TSF_d_ × Wet forest			-0.009 ± 0.013	0.167		
Horizontal diversity								
	Y + TSF_d_ × FT		0.00	0.95			0.55	0.65
		Year 2			-0.003 ± 0.004	0.119		
		TSF_d_			0.013 ± 0.004	<0.001		
		Wet forest			0.039 ± 0.007	<0.001		
		TSF_d_ × Wet forest			-0.010 ± 0.007	0.006		
	Y + TSF_d_ + FT		5.89	0.05			0.53	0.63
		Year 2			-0.002 ± 0.004	0.292		
		TSF_d_			0.010 ± 0.003	<0.001		
		Wet forest			0.039 ± 0.014	<0.001		
	Y + TSF_m_ + FT		21.61	0.00			0.46	0.63
		Year 2			0.000 ± 0.005	0.863		
		TSF_m_			0.077 ± 0.041	0.001		
		Wet forest			0.039 ± 0.016	<0.001		

Estimates associated with wet forest represent contrasts with dry forest, and estimates associated with Year 2 represent contrasts with Year 1. Models were ranked using Akaike’s information criterion corrected for small sample size (AIC_c_), and the three highest-ranked models per set are displayed with Akaike weights. Parameter estimates with 95% confidence intervals (CI) are displayed with their statistical significance (*P*). Two measures of fit are provided: marginal R^2^m (R^2^m) is the variance explained by fixed factors and conditional R^2^ (R^2^c) is the variance explained by both fixed and random factors.

## Discussion

Disturbance regimes are changing at unprecedented rates globally [63], and the consequences for FD are poorly known. In particular, there is a lack of information regarding associations between FD and measures of vegetation structure quantified at local scales of management relevance (but see [22]). To address this knowledge gap, we used landscape sampling units to investigate relationships between bird FD, vegetation diversity and measures of landscape TSF in two forest types. We found opposing responses of FD to vegetation diversity in wet and dry forest, but identified consistency in relationships between vegetation diversity and landscape TSF variables. We discuss the new insights arising from this work in the context of ecological management of fire-prone environments.

### Contrasting responses of FD to vegetation diversity

Findings were consistent with our prediction of contrasting responses of FD to vegetation diversity in different forest types. Responses of species richness followed classical expectations in dry forest [53,64], where it increased with vegetation diversity [65,66], and FRic and FDis followed the same pattern. Positive responses of species richness and FD to vegetation diversity are consistent with theory relating to environmental variation and coexistence. Variable environments are conducive to niche diversification because they offer greater opportunities for partitioning resources, and species are expected to coexist by minimising niche overlap. Structurally diverse vegetation is also expected to support a greater diversity of bird functional traits, and our results are consistent with this expectation in dry forest.

In theory, positive responses of FD to vegetation diversity are driven by multiple associations between individual traits and individual elements of vegetation structure, and the fourth-corner analysis revealed many such interactions in dry forest. Among the stronger interactions were positive responses of fruit-eating and mixed-diet birds to canopy cover, and a negative response of bark-foragers to midstory cover; presumably these relationships were driven by food availability. We lack explanations for several of the stronger interactions, such as the positive response of arboreal granivores to lower understory cover; clearly, many factors are likely to interact with vegetation structure in influencing the occurrence of species’ functional traits.

A patchwork of positive and negative responses of traits to vegetation structure was evident in both forest types; for example, while arboreal granivores responded positively to lower understory cover in dry forest, insect-eating and mixed-diet birds responded negatively. In wet forest, ground and understory foragers responded negatively to midstory cover, while builders of saucer-shaped nests responded positively. It is difficult to determine the relative importance of individual traits without detailed field observations over long periods [13], and therefore it is not possible to prioritise the conservation of particular attributes of vegetation structure. However, our results indicate that spatially variable vegetation structure is likely to support the largest number of bird functional traits.

Some trait-vegetation interactions were common to both forest types, but associations were generally weaker and less numerous in wet forest. Differences in the number and strength of trait-vegetation interactions in wet and dry forest are congruent with studies that show vegetation diversity is more important when productivity is lower and disturbance more frequent [36]. Further, our results were consistent with the expectation of a negative relationship between FD and vegetation diversity in productive wet forest. Bird species richness has been shown to decline in productive landscapes despite increases in vegetation diversity [36]. Huston’s dynamic equilibrium hypothesis proposes that in productive systems, a few species dominate communities in the absence of disturbance and competitively exclude other species [38,67]. The dynamic equilibrium hypothesis originally related to plant species, but it has since been supported by studies of forest birds [36,37]. To date, much research has focussed on the responses of species richness to vegetation diversity and productivity; some studies have also investigated responses of FD to vegetation diversity and productivity, but general patterns are yet to emerge [10,16,68]. For example, Seymour et al. [10] examined responses of species richness and FD to three vegetation types along an aridity gradient in Namibia. They detected increases in FD with increasing rainfall and vegetation structure, and suggested that the lack of a hump-shaped relationship was a consequence of high disturbance rates across the entire aridity gradient. To our knowledge, ours is the first empirical study of relationships between FD and vegetation structure to show congruence with Huston’s hypothesis.

The response of FEve to horizontal diversity presents a major exception to general trends; responses were opposite to those of species richness, FRic and FDis. In wet forest, while FRic and FDis decrease with increasing vegetation diversity, the distribution of species in functional space becomes more even [47], and the reverse pattern is apparent in dry forest. FEve is potentially a more meaningful measure of ecosystem function than other FD indices; it has been related to more efficient resource use in birds [18,69], which is consistent with the theory that evenness of species’ traits influences ecosystem processes independent of taxonomic richness [70].

### Vertical and horizontal diversity

Vertical diversity was the best predictor of four out of our five bird response variables, in accordance with many studies that show Foliage Height Diversity (the distribution of vegetation among vertical layers) is a principal driver of bird species diversity [53,71,72]. Recent studies have also sought to quantify horizontal diversity, which has been related to bird species richness at multiple scales [66,73]. We found that horizontal diversity was the most influential predictor of FEve, and although we lack a mechanistic explanation, it is clear that use of both variables in tandem can shed additional light on relationships between FD and vegetation diversity.

### Conclusions

Forests support a high proportion of global biodiversity [74], but the effects of disturbances such as fire on ecosystem function are largely unknown [31,32]. Identification of the key drivers of FD in fire-prone systems is required to guide management of forest landscapes, and to sustain ecosystem resilience [9,10]. Our study has shown stark contrasts in responses of FD to vegetation diversity within a 60,000-ha region comprising two broad forest types, highlighting the importance of tailoring fire management to local settings. Landscape TSF variables were very weakly related to vegetation diversity in wet forest, indicating that planned fire is unlikely to influence FD at the scale of our sampling units. However, in dry forest, the mean and diversity of TSF were positively correlated with vegetation diversity, indicating that it is possible for fire managers to manipulate FD. Elements of our results support findings of other studies that show older vegetation is disproportionately important to fauna [29,30]; however, our data also indicate that lower vegetation diversity is associated with increased FEve in dry forest. Theory and some empirical data show that FEve is particularly important to ecosystem function and resilience [9,18], but further work is required to elucidate the relative importance of FD metrics in our system. Validating assumptions that link individual traits to functions requires detailed field observations of many species over long time periods [13]. In the absence of field observations, FD provides a tractable means of understanding the influence of disturbance on ecosystem function. Several authors highlight the limitations of species richness as a measure of diversity (e.g. [75]) and emphasise the value of examining the responses of FD indices to environmental change [13,14]. In managed landscapes FD indices should be quantified at scales relevant to both the taxa of interest and to land management operations [15,18,22]. Given a lack of compelling evidence that one FD metric should be prioritised over others, we conclude that protection of older vegetation is important, and that controlled use of low-severity planned fire in dry forest may also sustain ecosystem function by enhancing different elements of FD.
